# Supplementary material for: An analysis of the spatial association between deforestation and agricultural field sizes in the tropics and subtropics
Source: PLoS One. 2019 Jan 30;14(1):e0209918. doi: 10.1371/journal.pone.0209918 (PMC6353091; doi:10.1371/journal.pone.0209918)
Supplement: S1 File — (DOCX) [file pone.0209918.s001.docx]

**S1 File**

**Supplementary Methods**

The following formula was used to define agricultural value:

$AR_{i}=\left(p_{ij}y_{ij}-c_{l}\right)-c_{T}$ (1)

where *p_ij_* is the price of crop *j* in cell *i*, *y_ij_* is the potential yield, *c_l_* is the labor cost approximated using the cost to plant and harvest the crop and *c_T_* are the transportation costs to bring the produce to the market in the nearest city. We considered the top 10 crops in terms of value and area in tropical and sub-tropical regions [[1](#_ENREF_1)]. This led to 18 crops: banana, bean, cassava, cocoa, coconut, coffee, cotton, cowpea, groundnut, maize, millet, oil palm, rice, rubber, sorghum, soybean, sugarcane, wheat. The individual maps of the 18 crops were overlaid together and masked with the spatial layers of deforestation and field size. The crop with the largest area in each cell was used to derive agricultural values. The spatial distribution of the crops were available for the year 2000 [[2](#_ENREF_2)]. In addition, we considered cattle head density as a proxy of livestock activities intensity [[3](#_ENREF_3)]. Transportation costs were approximated by summing the fuel costs and driver costs. To distinguish economic processes that were not dependent on market accessibility from those that were, we defined the variable “agricultural value” as the agricultural rent without considering transport costs and considered transportation costs separately to production costs in the statistical models.

The crop yield for the crops considered were represented using the average rain-fed potential yields from 1961 to 1990 [[4](#_ENREF_4)] except for the yield for rubber[[2](#_ENREF_2)]. The crops prices were obtained from FAOSTAT and expressed in USD/ton[[1](#_ENREF_1)]. We collected the prices of 11 years from 1995 to 2005 and converted them to dollars of the year 2005 using GDP deflators. We then took the average to minimize the influence of price fluctuations. For some countries such as Afghanistan, Cuba and Myanmar there were no price available in USD. We instead used the local currencies and converted them to USD using purchasing power parity tables.

The total labour cost was calculated for each of the 18 crops as the product of the hourly wage, the number of man-hours required to produce one ton of the crop and the crop’s yield in tons per hectare:

$Labour Cost=Wage\times Man\_hour per tonne\times Yield$ (1)

The hourly wage was calculated from daily wages [[5](#_ENREF_5)] assuming 8 working hours a day. The man hours need to produce 1 tonne of crop is estimated using estimated and averaged using data from various literature[[6-17](#_ENREF_6)]. The driver cost was calculated as the product of the hourly wage for one way trip, the number of hours needed to travel to the nearest city and the number of trucks needed to carry the yield of 1 hectare of the crop if the trucks are fully loaded:

$Driver Cost=Wage\times Accessibility\times\frac{\mathrm{Yield}}{Max load}$ (2)

The fuel cost (for any crop) was calculated as the product of the fuel price (by country) and the amount of fuel to carry a ton of crop one mile. This was estimated by multiplying the crop’s yield per ha, the distance travelled and the average fuel consumption of a truck.

$Fuel Cost=Yield\times Average Speed\times Accessibility\times$ (3)

$Fuel Price\times Fuel Consumption per ton\_mile$

We assumed the average speed and fuel consumption of a heavy truck of class 6 (beverage/ single axle/ school bus/ rack truck) with typical maximum load capacity of 11500 pounds. All prices were converted to international dollars of the year 2005. For countries with no data, we used the mean value of the data from all available countries as an estimate.

The final variables agricultural rent, agricultural value and transportation cost were expressed in 2005 USD/ha. We used the individual crops agricultural rent and agricultural values in two ways in our model: either using the value of the dominating crop at a cell or using a weighted mean of the values of all crops.

**Supporting Tables**

Table A. Summary of data sources used, type of variables and their descriptive statistics.

| **Data** | **Value/ Unit** | **Descriptive statistics (mean and standard deviation)** | **Source** |
| --- | --- | --- | --- |
| Field size maps (for 2005) | 10-40 (small to very large) | 22.73 (7.72) | [[18](#_ENREF_18)] |
| Forest loss map (2000-2001) | 0-900 (number of 1sec∙1sec cells with forest loss inside a 30sec∙30sec cell) | — | [[19](#_ENREF_19)] |
| Accessibility (2000) | minutes to nearest big city | 302.61 (445.43) | [[20](#_ENREF_20)] |
| Population density | persons/ km2 | 94.44 (321.23) | [[21](#_ENREF_21)] |
| Protected Area | Polygons/points representing protected regions | 5.61 % of points are in protected areas; 94.39 % are not | [[22](#_ENREF_22)] |
| Cattle head count | heads per km2 | 21.62 (38.43) | [[3](#_ENREF_3)] |
| **Variables used to calculate Agricultural Value** | | | |
| Potential Yields* | ton/ha | — | [[4](#_ENREF_4)] |
| Crop prices | USD/ton | — | [[1](#_ENREF_1)] |
| Daily wage | USD/hour | 25.15 (19.305) | [[5](#_ENREF_5)] |
| Fuel Price | USD/litre | 0.62 (0.25) *** | [[23](#_ENREF_23)] |
| Truck characteristics | Maximum capacity : 11,500 pounds; average speed: 65 km/h; fuel consumption: 77.22 litres per thousand ton-miles | — | [[24](#_ENREF_24)] |
| Man-hours required to produce one ton of a given crop | hours/ton | 137.08  (93.12) | [[6-17](#_ENREF_6)] |
| Harvested area in year 2000 for 18 crops | fraction of five-minute grid cell harvested area ** | 0.00885 (0.0383) | [[2](#_ENREF_2), [25](#_ENREF_25)] |

* We performed a spatial join of wetland and dryland rice yield (average in overlapping regions); pearl and foxtail millet

** A grid cell may be more than 1.0 or 100% due to multiple harvests per year.

*** Price adjusted to 2005 USD

Table B. Characteristics of the models considered using the information theoretic approach. All models used field size as the independent variable, and included country as a random effect.

| Model Rank | Protected Area | Agricultural Value | Accessibility | Cattle | Population Density | Other terms | Spatial Correlation Structure | AIC |
| --- | --- | --- | --- | --- | --- | --- | --- | --- |
| 1 | -0.047372 | 0 | 0 | 0 | 0 | 0 | Rational Quadratic | 103625.2 |
| 2 | 0 | -0.03837 | 0 | 0 | 0 | 0 | Rational Quadratic | 103626.1 |
| 3 | -0.048441 | -0.039562 | 0 | 0 | 0 | 0 | Rational Quadratic | 103630.4 |
| 4 | 0 | 0 | 0.000777 | 0 | 0 | 0 | Rational Quadratic | 103633 |
| 5 | -0.048242 | 0 | 0.001025 | 0 | 0 | 0 | Rational Quadratic | 103637.4 |
| 6 | 0 | 0 | 0 | -0.000048 | 0 | 0 | Rational Quadratic | 103638.2 |
| 7 | -0.049267 | -0.039388 | 0.000978 | 0 | 0 | 0 | Rational Quadratic | 103642.6 |
| 8 | 0 | 0 | 0 | 0 | 0.000002 | 0 | Rational Quadratic | 103642.7 |
| 9 | -0.047513 | 0 | 0 | -0.000052 | 0 | 0 | Rational Quadratic | 103642.7 |
| 10 | 0 | -0.038326 | 0 | -0.000046 | 0 | 0 | Rational Quadratic | 103643.6 |
| 11 | -0.048578 | -0.039517 | 0 | -0.000051 | 0 | 0 | Rational Quadratic | 103647.9 |
| 12 | 0 | -0.038389 | 0 | 0 | 0.000002 | 0 | Rational Quadratic | 103648 |
| 13 | 0 | 0 | 0.000777 | -0.000048 | 0 | 0 | Rational Quadratic | 103650.5 |
| 14 | -0.017457 | 0 | 0 | -0.000020 | 0 | Protected Area and Cattle Interaction  -0.002196 | Rational Quadratic | 103651.3 |
| 15 | -0.048384 | 0 | 0.001025 | -0.000052 | 0 | 0 | Rational Quadratic | 103654.9 |
| 16 | 0 | 0 | 0.000792 | 0 | 0.000002 | 0 | Rational Quadratic | 103655 |
| 17 | 0 | -0.038182 | 0.000727 | -0.000046 | 0 | 0 | Rational Quadratic | 103655.9 |
| 18 | -0.018474 | -0.039690 | 0 | -0.000019 | 0 | Protected Area and Cattle Interaction  -0.002200 | Rational Quadratic | 103656.5 |
| 19 | -0.049404 | -0.039344 | 0.000979 | -0.000051 | 0 | 0 | Rational Quad | 103660.1 |
| 20 | 0 | -0.038246 | 0.000742 | 0 | 0.000002 | 0 | Rational Quadratic | 103660.3 |
| 21 | -0.018342 | 0 | 0.000974 | -0.000021 | 0 | Protected Area and Cattle Interaction  -0.002191 | Rational Quadratic | 103663.5 |
| 22 | -0.049269 | -0.039409 | 0.000994 | 0 | 0.000002 | 0 | Rational Quadratic | 103664.5 |
| 23 | -0.01931 | -0.039525 | 0.000927 | -0.000019 | 0 | Protected Area and Cattle Interaction  -0.002196 | Rational Quadratic | 103668.7 |
| 24 | -0.048569 | -0.039535 | 0 | -0.000051 | 0.000002 | 0 | Rational Quadratic | 103669.8 |
| 25 | 0 | 0 | 0.000791 | -0.000047 | 0.000002 | 0 | Rational Quadratic | 103672.5 |
| 26 | -0.048385 | 0 | 0.001039 | -0.000052 | 0.000002 | 0 | Rational Quadratic | 103676.8 |
| 27 | 0 | -0.038203 | 0.000742 | -0.000046 | 0.000002 | 0 | Rational Quadratic | 103677.8 |
| 28 | -0.018470 | -0.039706 | 0 | -0.000019 | 0.000002 | Protected Area and Cattle Interaction  -0.002199 | Rational Quadratic | 103678.4 |
| 29 | -0.049406 | -0.039364 | 0.000994 | -0.000051 | 0.000002 | 0 | Rational Quadratic | 103682 |
| 30 | -0.132777 | -0.043708 | 0.001085 | -0.000056 | 0.000002 | Protected Area and Agricultural Value  0.053631 | Rational Quadratic | 103684 |
| 31 | -0.018348 | 0 | 0.000987 | -0.000020 | 0.000002 | Protected Area and Cattle Interaction  -0.002191 | Rational Quadratic | 103685.4 |
| 32 | -0.019319 | -0.039545 | 0.000941 | -0.000019 | 0.000002 | Protected Area and Cattle Interaction  -0.002195 | Rational Quadratic | 103690.7 |
| 33 | -0.027005 | -0.039533 | 0.001405 | -0.000051 | 0.000002 | Protected Area and Accessibility  -0.003985 | Rational Quadratic | 103692.2 |
| 34 | -0.049500 | -0.033436 | 0.003072 | -0.000050 | 0.000002 | Agricultural Value and Accessibility  -0.001683 | Rational Quadratic | 103693.8 |
| 35 | -0.049161 | -0.026674 | 0.001006 | -0.000051 | 0.000002 | Quadratic Term for Agricultural Value  -0.000860 | Rational Quadratic | 103695.2 |
| 36 | -0.049688 | -0.045031 | 0.000990 | -0.000312 | 0.000002 | Agricultural Value and Cattle Interaction  0.000192 | Rational Quadratic | 103698.3 |
| 37 | -0.038834 | -0.039620 | 0.000957 | -0.000051 | 0.000006 | Protected Area and Population Interaction  -0.000136 | Rational Quadratic | 103698.6 |
| 38 | -0.049466 | -0.039757 | 0.000991 | -0.000051 | -0.000004 | Agricultural Value and Population Interaction 0.000004 | Rational Quadratic | 103704.4 |
| 39 | -0.047786 | 0 | 0 | 0 | 0 | Quadratic Terms for Agricultural Value 0.001368, Accessibility 0.000021, Cattle 0.000000, and Population Density 0.000000 | Rational Quadratic | 103726.9 |
| 40 | -0.049032 | -0.026718 | 0.000864 | -0.000206 | -0.000007 | Quadratic Terms for Agricultural Value 0.000859, Accessibility 0.000004, Cattle 0.000000, and Population Density 0.000000 | Rational Quadratic | 103782.1 |
| 41 | -0.054155 | -0.045233 | 0.001413 | -0.000088 | -0.000005 | 0 | Exponential | 107548.1 |
| 42 | -0.052385 | -0.046814 | 0.001455 | -0.000087 | -0.000004 | 0 | Spherical | 107679.7 |
| 43 | -0.059839 | -0.040293 | 0.002111 | -0.000023 | 0.000004 | 0 | Gaussian | 109166.3 |
| 44 | Did not converge | Did not converge | Did not converge | Did not converge | Did not converge | 0 | Linear | Did not converge |

**Supporting Figures**

Figure A. Images provided to Geo-Wiki users as a reference of large, medium, small and very small field categories. The image was provided by Fritz and colleagues [[18](#_ENREF_18)].


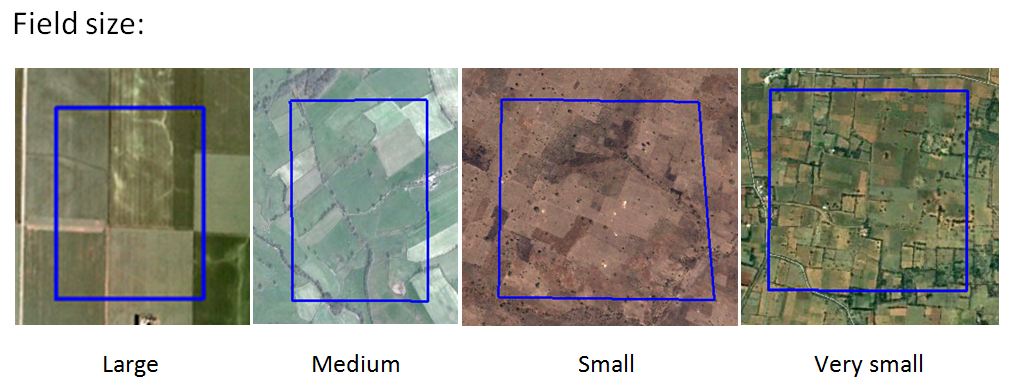


Figure B. Scatter plot of model variables. The scatter plots of field size against the predictors imply that a linear relationship is enough to explain the effect of the predictors on the field size, and thus we focused on linear- mixed effect model.

Figure C. Scatterplot of the changes in coefficients and standard errors of the two top models when varying the definition of deforestation from 1 sub-cell out of 900 in a 30 second by 30 second cell up to 800 sub-cells out of 900. Beyond 800, the model for agricultural value will no longer converge, and beyond 550, the model for protected area will no longer converge.


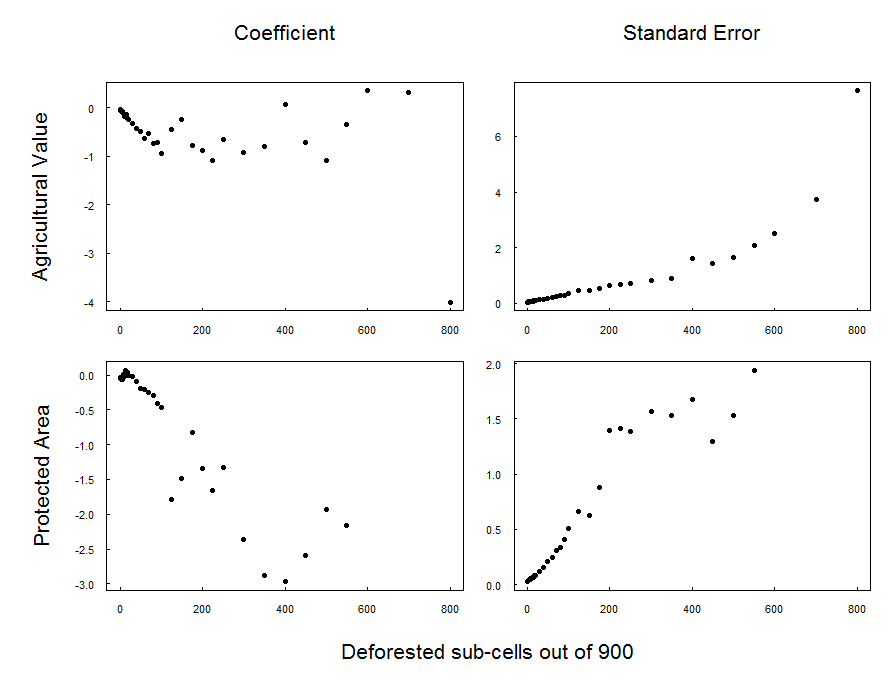


Figure D. Scatterplot showing the change in the size of our data set when increasing the definition of deforestation from 1 sub-cell out of 900 in a 30 second by 30 second cell up to 900 sub-cells out of 900


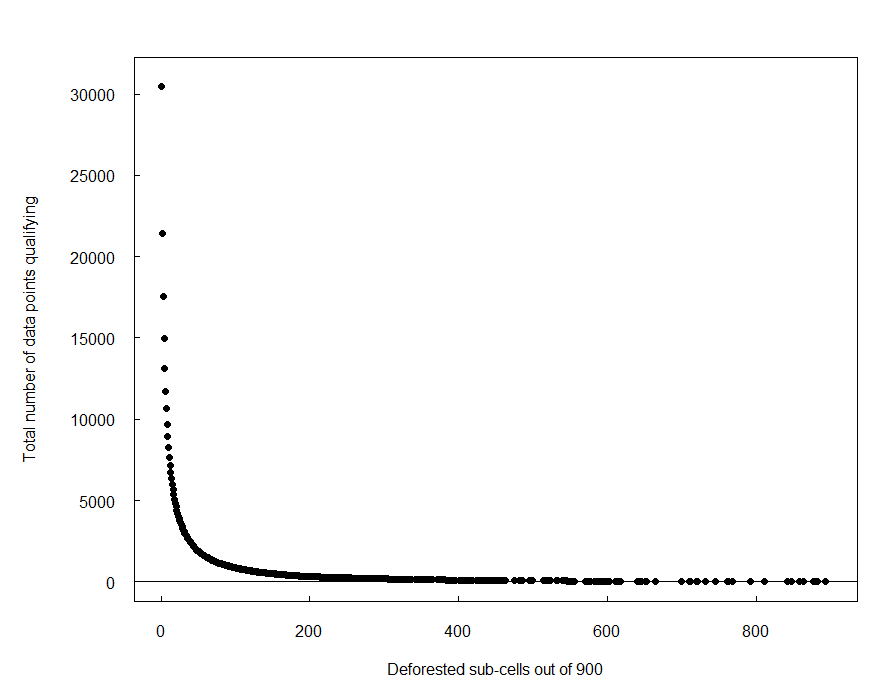


Figure E. Bar plot of distribution of field sizes in deforested areas in each country, ordered by average field size for each country. A field size of 10 is “very small”, 20 is “small”, 30 is “medium”, and 40 is “large”.

**References**

1. FAO. FAOSTAT. Food and Agriculture Organization of the United Nations. Available at: <http://faostat.fao.org/site/342/default.aspx>. 2010.

2. Monfreda C, Ramankutty N, Foley JA. Farming the planet: 2. Geographic distribution of crop areas, yields, physiological types, and net primary production in the year 2000. Global Biogeochem Cycles. 2008;22(1):GB1022. doi: 10.1029/2007gb002947.

3. FAO. Livestock densities. Gridded Livestock of the World (GLW). Food and Agriculture Organization of the United Nations. Animal Production and Health. Accessed at: <http://www.fao.org/ag/againfo/resources/en/glw/glw_dens.html>. 2014.

4. International Institute for Applied Systems Analysis. GAEZ v3.0 Global Agro-ecological Zones. IIASA. Accessed at: <http://webarchive.iiasa.ac.at/Research/LUC/GAEZv3.0/>. 2014.

5. ILO. International Labor Office Department of Statistics. United Nations International Labour Organization. Available at: <http://laborsta.ilo.org/default.html>. 2011.

6. Van Damme P, Sampers W, Pauwels F. Socio-Economic Aspects Of The Intensive Growing Of Cowpeas (Vigna Unguiculata (L.) Walp.) In Kano, Northern Nigeria. AFRIKA FOCUS. 1986;2(2):171-94.

7. Singh DV. Production and marketing of off-season vegetables: Mittal Publications; 1990.

8. Maleka P. An application of Target MOTAD Modelto crop production in Zambia: Gwembe Valley as a case study. Agricultural Economics, . 1993;9:15-35.

9. Reynolds SG. Pasture-cattle-coconut systems: Food and Agriculture Organization of the United Nations.; 1995.

10. Nobre Lages V. Resource-use patterns: The case of coconut-based agrosystems in the coastal zones of Kerala, India.1996.

11. Nyende P, Tenywa JS, Oryokot J, Kidoido M. Weed profiles and management assessment for increased finger millet production in Uganda. 2001.

12. Sidhu HS. Production conditions in contemporary Punjab agriculture. JPS. 2005;12(2).

13. Karivaradaraaju TV. Processing of Cotton Seed at SIMA CD & RA. 2007.

14. Abdullahi A, Tsowa M. Economics of cowpea production under small-scale cowpea enterprise in Agricultural Zone I of Niger State, Nigeria. IOSR Journal of Agriculture and Veterinary Science 2010;7(4):84-94.

15. Hewavitharane HVC, Warnakulasooriya HU, Wajira Kumara GBS. Constraints To Expansion Of Cowpea And Mungbean Under Rain-Fed Farming In Anuradhapura District. Annals of the Sri Lanka Department of Agriculture. 2010.

16. Faki H, Ismail M, Board SG, Barakat S, editors. Some indicators for wheat production prospects in Sudan. Wheat in Heat-stressed Environments: Irrigated, Dry Areas, and Rice-wheat Farming Systems: Proceedings of the International Conferences: Wheat in Hot, Dry, Irrigated Environments, Wad Medani, Sudan, 1-4 February 1993, Wheat in Warm Area, Rice-wheat Farming Systems, Dinajpur, Bangladesh, 13-15 Feburary [sic] 1993; 1994: CIMMYT.

17. Waterbury J. The Senegalese peasant: How good is our conventional wisdom? The political economy of risk and choice in Senegal Frank Cass, Londres. 1987.

18. Fritz S, See L, McCallum I, You L, Bun A, Moltchanova E, et al. Mapping global cropland and field size. Global Change Biology. 2015.

19. Hansen MC, Potapov PV, Moore R, Hancher M, Turubanova SA, Tyukavina A, et al. High-Resolution Global Maps of 21st-Century Forest Cover Change. Science. 2013;342. doi: 10.1126/science.1244693.

20. Nelson A. Accessibility model and population estimates. background paper and digital files prepared for the World Development Report. 2009.

21. McGranahan G, Balk D, Anderson B. Low Elevation Coastal Zone (LECZ) Urban-Rural Population Estimates, Global Rural-Urban Mapping Project (GRUMP), Alpha Version. Palisades, NY: NASA Socioeconomic Data and Applications Center (SEDAC). 2007.

22. WDPA Consortium. World database on protected areas. World Conservation Union and UNEP-World Conservation Monitoring Centre, New York, New York, USA. 2004.

23. World Bank. Pump price for gasoline (US$ per liter). Accessed at: <http://data.worldbank.org/indicator/EP.PMP.SGAS.CD>. 2016.

24. Davis SC, Diegel SW, Boundy RG, Moore S. 2014 Vehicle Technologies Market Report. Oak Ridge National Laboratory, 2014.

25. Ramankutty N, Evan AT, Monfreda C, Foley JA. Farming the planet: 1. Geographic distribution of global agricultural lands in the year 2000. Global Biogeochemical Cycles. 2008;22(1).
