# Supplementary material for: Correction: An analysis of the spatial association between deforestation and agricultural field sizes in the tropics and subtropics
Source: PLoS One. 2019 Feb 14;14(2):e0212596. doi: 10.1371/journal.pone.0212596 (PMC6375699; doi:10.1371/journal.pone.0212596)
Supplement: S2 File — (PDF) [file pone.0212596.s002.pdf]

PLOS ONE

RESEARCH ARTICLE

An analysis of the spatial association between deforestation and agricultural field sizes in the tropics and subtropics

Doan K. D. Dang[☯], **Amy C. Patterson**[☯], **Luis R. Carrasco***

Department of Biological Sciences, National University of Singapore, Singapore, Republic of Singapore

☯ These authors contributed equally to this work.
* dbsctlr@nus.edu.sg

Check for updates

OPEN ACCESS

Citation: Dang DKD, Patterson AC, Carrasco LR (2019) An analysis of the spatial association between deforestation and agricultural field sizes in the tropics and subtropics. PLoS ONE 14(1): e0209918. https://doi.org/10.1371/journal.pone.0209918

Editor: Julia A. Jones, Oregon State University, UNITED STATES

Received: July 12, 2018

Accepted: December 13, 2018

Published: January 30, 2019

Copyright: © 2019 Dang et al. This is an open access article distributed under the terms of the Creative Commons Attribution License, which permits unrestricted use, distribution, and reproduction in any medium, provided the original author and source are credited.

Data Availability Statement: All relevant data are within the paper and its Supporting Information files.

Funding: L.R.C., A.C.P. and D.K.D.D. were supported by the Ministry of Education of Singapore under the Tier 2 grant MOE2015-T2-2-121.

Competing interests: The authors have declared that no competing interests exist.

Abstract

Tropical deforestation is one of the most pressing threats to biodiversity, and substantially reduces ecosystem services at the global scale. Little is known however about the global spatial distribution of the actors behind tropical deforestation. Newly available maps of global cropland field size offer an opportunity to gain understanding towards the spatial distribution of tropical deforestation actors. Here we use a map of global cropland field size and combine it with maps of forest loss to study the spatial association between field size and deforestation while accounting for other anthropogenic and geographical drivers of deforestation. We then use linear mixed–effects models and bootstrapping to determine what factors affect field sizes within deforested areas across all countries in the global tropics and subtropics. We find that field size within deforested areas is largely determined by country-level effects indicating the importance of socio-economic, cultural and institutional factors on the distribution of field sizes. Typically, small field sizes appear more commonly in deforested areas in Africa and Asia while the association was with larger field sizes in Australia and the Americas. In general, we find that smaller field sizes are associated with deforestation in protected areas and large field sizes with areas with lower agricultural value, although these results have low explanatory power. Our results suggest that the spatial patterns of actors behind deforestation are aggregated geographically which could help target conservation and sustainable land-use strategies.

Introduction

Tropical forests are the most biodiverse terrestrial biome on Earth, and are instrumental in 15 out of the 25 global biodiversity hotspots [1]. Besides their vital role as habitat for many species, tropical forests also provide ecosystem services such as carbon sequestration, disease regulation, increased rainfall and provide subsistence food and resources for tropical communities [2–4]. However, tropical forests are increasingly under threat. Tropical deforestation is happening at rapid rates with 730 thousand square kilometers of tropical forests lost globally from 2000 to 2012 [5]. This loss, if left unchecked, will lead to substantial declines in the

abundance of many species, possibly even to large-scale extinctions [6], as well as the depletion of valuable ecosystem services which could hinder the development options of future generations [7].

The primary cause of rapid tropical deforestation is the increasing demand for timber, biofuels, and agricultural products. For instance, 55% of new agricultural land in the 1980s and 1990s was at the expense of intact tropical forests [8]. Growing demand caused by the rising global population is exacerbated by an increasing per capita demand due to economic growth in developing countries. This poses a challenge to tropical forest conservation since tropical forests occupy most of the remaining unused land suitable for agriculture [8]. The pressure to convert forest to agriculture is exemplified by the main drivers of deforestation. These have been shown in Latin America to be cropland and pastureland expansion between 2001 and 2013 [9], and in Africa to be agricultural and fuelwood demands [10].

Despite our knowledge of the large scale drivers of tropical deforestation and its large scale environmental consequences, we lack, at the global scale, knowledge of the actors that are behind most deforestation. One potential way to obtain an approximate solution to the problem is to use field sizes as a proxy for actors, whereby small field sizes would be associated to smallholders and large field sizes associated to large agri-businesses. For instance, field sizes from smallholders are typically below two hectares [11]. Field size maps are thus important because they could be used, with inherent caveats, as a surrogate for farm size [12], income [13] and level of mechanization [14]. Field sizes could thus be a good proxy to advance towards differentiating between smallholder agriculture and large agri-businesses. The link between field size data and actor identification thus opens an opportunity to learn about deforestation and support land-use and conservation interventions. This is because of the different constraints and capabilities associated with smallholders and large agri-businesses. By knowing which actor is responsible of deforestation in each location can have useful applications for the design and spatial targeting of conservation interventions. However, a global classification of actors behind deforestation has remained elusive with approximations using size of land clearings [15] and identification of actors occurring only for individual countries [16, 17]. A dramatic improvement in this line of enquiry comes from a recent global map identifying types of drivers of deforestation including commodity agriculture, shifting agriculture, forestry and wildfires [18].

The recent availability of the first global field size map [19] presents an unprecedented opportunity to contribute further to characterize and monitor agricultural activities globally. We use this new dataset to globally characterize the conditions associated with the occurrence of different field sizes, thereby examining the factors that influence a potential proxy for the distribution of smallholders and large agri-businesses. Specifically, we aim to ascertain: (i) the countries and regions in which large field sizes are associated with deforestation; and (ii) the factors that spatially predict field size in deforested areas. We hypothesize that field sizes within recently deforested areas have spatial patterns that are distinctive in each country and that can be explained by accessibility and higher agricultural values. We do this by studying deforestation from 2000 to 2001 and field sizes replacing forests in 2005.

Methods

Data collection

We compiled spatial data using Geographic Information Systems (GIS) for our main variables of field size and deforestation as well as for variables influencing field size within deforested areas. These included the potential rents for agricultural crops and cattle, (defined as the rent that is obtained under potential yields after closing yield gaps), country boundaries, protected

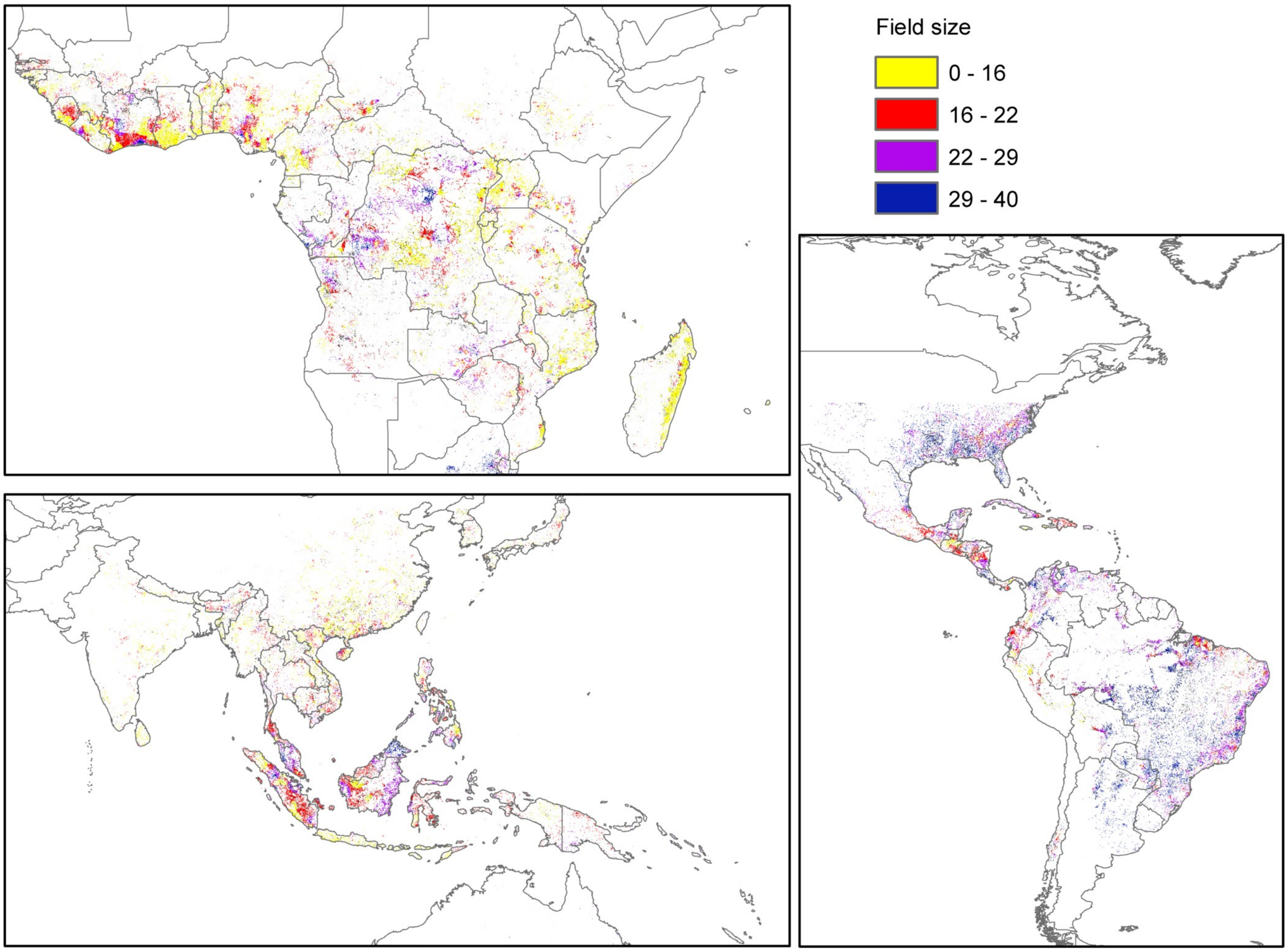

Fig 1. Distribution of field sizes within deforested land from 2000 to 2001 for which agricultural information exists. a) Africa; b) Asia; and c) Americas. The map was built through overlaying a map of field sizes [19] and maps of deforestation [5]. Those cells that contained information on deforestation and field size and at least one crop were selected.

https://doi.org/10.1371/journal.pone.0209918.g001

areas, accessibility, and population density (Table A in S1 File describes the variables and their descriptive statistics). The spatial scope of the analysis was tropical and subtropical regions defined as latitudes from 40 degrees South to 40 degrees North.

The maps were overlaid using ArcMap 10.2.1. We further restricted our dataset to include only areas that were deforested between 2000 and 2001 and excluded all other areas that presented no deforestation between 2000 and 2001. We chose 2000–2001 for two reasons: (i) this was the earliest year with high-resolution deforestation information available that occurred before the field size map was created (2005), allowing us to assume that enough time had passed since deforestation to the establishment of the agricultural fields associated to the cleared land; and (ii) it was *circa* the year 2000 for which agricultural values were calculated, and which best matched with our other data sets. We then use systematic sampling to select a set of 35,175 30 second by 30 second cells (Fig 1). Information for the other variables considered was extracted to this set of cells. When two different field sizes occurred within a deforested cell, the mean of the field size values was calculated.

Variables considered

—We use field size within areas deforested in the year 2000 as the dependent variable. This was obtained from the first ever global map of field size, which was developed for the year 2005 using interpolation and data from Geo-Wiki, a citizen science project using satellite images [19]. Fields were categorized from 1 (very small) to 4 (large) in 13,963 unique locations. All images were ranked on the same scale, resulting in a map that is globally consistent even between different countries. These data were rescaled to a range of 10 (very small) to 40 (large) and interpolated using inverse weighted distance treating the ordinal values as a continuum [19]. Fritz et al. [19] provided Geo-Wiki users with sample pictures (Figure A in S1 File) for them to classify the field size in 1km·1km Google Earth images. Given that this was a qualitative process, it is not possible to ascertain with precision the correspondence between the categories large, medium, small and very small to actual areas. A rough visual estimate however would correspond to an average of >25, 3.3, 1 and 0.5 hectares per field for each category respectively. The final map restricted the field sizes to a range of 10 to 40 using only integers. The map was validated by experts for quality, and a Spearman's rank correlation of 0.78 was found between the expert scores and the final map.

—The forest loss map was constructed using the original version of the deforestation maps created by Hansen et al. using satellite imagery [5] with 1 second·1 second resolution. To make the deforestation map have the same coarser resolution as the field size map (30 seconds), we aggregated the 1 second·1 second cells into 30 second·30 second cells classifying the aggregated cell as deforestation if at least one of the disaggregated cells was deforested in this time period. We did this aggregation using the function "aggregate" in ArcGIS and the sum as the criterion of aggregation. We later assessed the influence on the results of other thresholds for classification of deforestation (see "*Robustness analyses*") by varying the deforestation threshold from 1 out of 900 cells to 800 out of 900. We only used deforestation data from 2000 to 2001.

—Protected areas were expected to influence deforestation dynamics and were included in the model [20]. We obtained worldwide protected area maps from the World Database of Protected Areas [21].

—Accessibility was considered in the model because it affects transport costs, thus limiting commercial agricultural activities and affecting the occurrence of illegal logging [22]. A global map of accessibility, created for the year 2000 by the World Bank's World Development Report in 2009, was employed [23]. It expresses accessibility as time necessary to travel to a city of at least 50,000 habitants.

—Population density was included in the model. This was done as it was expected to relate to crop type and type of farm size [24].

—Agricultural rent was expected to increase the probability of deforestation [25] and to be associated with specific farm sizes [26]. We calculated the agricultural value of a crop in a cell i as the revenue of the crop minus the production costs without including transport costs (details of the calculation are available in S1 File as Supplementary Methods).

Statistical modeling

We created a scatter plot to check for non-linear relationships between field size and the covariates (Figure B in S1 File). Accessibility and transportation costs were highly correlated as one variable was used to estimate the other. Similarly, agricultural rent and agricultural value were correlated. To avoid problems of multicollinearity, these variables were not included simultaneously in the proposed models. The rest of the explanatory variables did not show any clear correlation and we included all of them in the models (Figure B in S1 File).

We fitted different linear mixed-effects models using the package nlme in the R statistical environment [27, 28] to explore the relationship between the field size and the explanatory variables (Table B in S1 File). R-squared values were calculated using the package MuMIn. As the data may not be independent due to common country effects such as government or socio-political factors not accounted for in the models, we employed a random country intercept component to capture the differences among countries. We also considered different spatial correlation structures in the models proposed (Table B in S1 File). We proposed 43 different models with varying spatial autocorrelation structures, different combinations of our explanatory variables, and different interactions and quadratic terms. Models were ranked according to their Akaike Information Criterion (AIC) using an information theoretic approach [29].

Specifically, the linear mixed-effects models with random intercept by country had the form:

$$Y_{ij} = X_{ij}\beta + c_i + \eta_{ij} \quad (1)$$

where Y_{ij} is the response variable, field size of the jth cell of the ith country; $c_i \sim N(0, \sigma_c^2)$ is the random intercept for country i and is assumed to follow a normal distribution with mean zero and variance σ_c^2. The errors η_{ij} are assumed to be independent and normally distributed with variance σ_c^2. c_i and η_{ij} are assumed to be independent. X_{ij} is the vector of fixed effects values for observation j in country i. Diagnostic plots were used to verify that the models did not present problems of heteroscedasticity and that they conformed to the assumptions of normality.

Robustness analyses

Due to the fact that the field size map on which we based our analyses was made using crowd-sourced data and interpolation, it entailed high uncertainty. To evaluate the influence of this uncertainty, we used a bootstrap analysis to evaluate the robustness of our results to uncertainty in this dataset. We used a data set employed for the validation of the field size data in which experts rate randomly chosen points using the same 1 (very small) to 4 (large) scale used by the Geo-Wiki crowdsourcing website [19]. Using the expert values to represent the true values of field size, we estimated an uncertainty distribution of expert classifications for points that had a classification in the map of 1, 2, 3 and 4 respectively. These distributions reflected the variability of the experts when classifying each size of fields. We then used these expert uncertainty distributions to modify the field size values in the map used for our analysis, i.e. we reproduced potential changes in the classification of the values that could have emerged due to uncertainty in expert classfication. We did this for each point in our dataset for each bootstrap run, by randomly selecting ten expert scores from the uncertainty distribution corresponding to the field size map value observed at that point and summing them to obtain a score to use in the model. This resulted in the same 10 to 40 integer-only scale present in the field size map.

We selected the statistical models which were within an AIC value of 2 from the model with the lowest AIC value. We then ran these 2 models 100 times, replacing the original field sizes with scores from the expert validation dataset as described above. For each run we used model weighting based on the AIC score to average the two models. After all of the runs, we determined average parameter values and constructed bootstrapped confidence intervals for each parameter (Table 1).

Besides the effects of uncertainty in the field size map on our model results, we also tested the sensitivity of our parameter estimates to our definition of deforestation. While we considered a cell deforested if at least one 1 second by 1 second sub-cell was deforested out of a 30 second by 30 second cell, we also raised our defined cutoff for deforestation and observed the

Table 1. Relationship between field size, protected area, and agricultural value resulting in the top averaged model. Protected area is a binary variable stating whether or not an observation is in a protected area. The results shown are from the bootstrapped weighted average of the two models that are within an AIC value of 2 from the model with the lowest AIC. The coefficient for protected area is robust given that the confidence interval does not include zero, while the coefficient for agricultural value is not robust.

Variable	Mean Value	Median Value	95% Confidence Interval Lower Bound	95% Confidence Interval Upper Bound
Intercept	27.95	27.95	27.82	28.08
Protected Area	-0.04	-0.03	-0.09	-0.006
Agricultural value	-0.015	-0.007	-0.113	0.056

https://doi.org/10.1371/journal.pone.0209918.t001

effect on parameter estimates and standard errors for our top two models (Figures C and D in S1 File).

Results

When overlaying the deforestation and field size maps for tropical and subtropical regions, two main trends became apparent. Large field sizes are more commonly distributed in deforested land in countries in Latin America, East Asia and Australia (Figs 1 and 2), with the largest field sizes occurring in Australia, Argentina, and Uruguay. By contrast, smaller field sizes tended to occur within deforested land in Africa and Southeast Asia (Figs 1 and 2), with the smallest average field sizes occurring in Burundi, Madagascar, and Rwanda.

Following a similar pattern to the raw distribution of field sizes across geographic regions, the random effects of the models with regards to individual countries corresponded well with

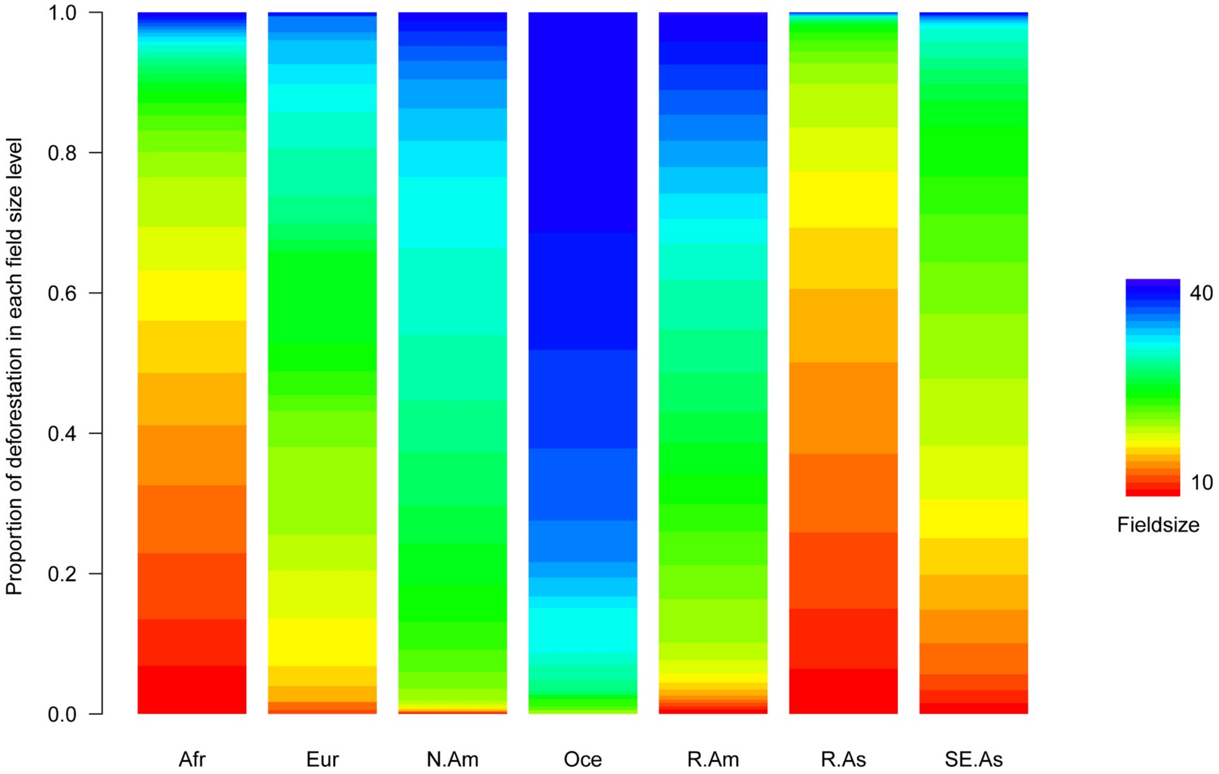

Fig 2. Distribution of cells deforested between 2000 and 2001 by field size across major regions (Figure E in S1 File shows a breakdown for the individual countries analyzed). Oceania is dominated in area by Australia. Afr: Africa, Eur: Europe, N.Am: North America, Oce: Oceania, R.Am: rest of America, SE.As: Southeast Asia R.As: rest of Asia.

https://doi.org/10.1371/journal.pone.0209918.g002

our expectations. Most of the variance in our models was explained by the random effect of country, indicating the importance of each country's socio-economic, cultural, and institutional factors in determining the field sizes behind deforestation (R-squared for random effects was 0.69). The estimates of the random intercepts by country indicated that tropical areas deforested in countries with a large tradition of agricultural exports such as Australia, Argentina, Uruguay, Paraguay, the United States and Brazil would be expected to have larger field sizes on average (Fig 3). At the other end of the spectrum, nations predominantly from Africa and Asia such as Burundi, Madagascar, Ethiopia, Bangladesh, India, and Laos were expected to have in comparison smaller field sizes associated with deforestation (Fig 3). It therefore appears that field sizes associated with deforestation follow specific regional patterns. For the most part, this pattern matched what would be expected given the raw distribution of field sizes in deforested areas in each region (Fig 2 and Figure E in S1 File).

Only one model had a difference of less than 2 AIC value from the model with the lowest AIC (Table B in S1 File). Of these top two models, one used protected area status alone as an independent variable, and the other used agricultural value alone. Lower field sizes were associated with protected areas, suggesting that deforestation within protected areas is generally driven by smallholders (Table 1) once country effects are controlled for (R-squared for fixed effects was $3.6 \cdot 10^{-6}$). Larger field sizes were associated on the other hand with lower agricultural value, which could correspond to large concessions to agri-business corporations which are often granted in intact forests, well beyond the traditional agricultural regions (R-squared for fixed effects was $6.4 \cdot 10^{-5}$) (Table 1). However, caution must be taken in the interpretation of the fixed effects of these two model results because the variance explained by these two variables was very low, much lower than the variance explained by the random effects of country.

Other variables that we hypothesized would help explain the distribution of field sizes in deforested areas were not included in our final models as selected by information theory. While accessibility was expected to be a major player in where large corporations and large field sizes could expand, it should be noted that contemporary deforestation frontiers occur in relatively similarly inaccessible areas, which may undermine the capacity of the model in observing an effect. Population density was also a promising potential predictor of field sizes, however within countries it mostly reveals where cities are located which is not where deforestation is occurring. Population differences, types of agricultural systems and urbanization patters may certainly be associated with different field sizes, such as the smaller field sizes in countries with high population density like China and India. However in our models this variation seems to have been explained by the random effects at the country level rather than the spatially explicit variables (Fig 3). Besides protected areas and agricultural value, we were also unable to find support for any interactions between explanatory variables or quadratic terms.

Through our bootstrap analysis, we found that the association of protected areas with smaller field sizes was robust to the uncertainty contained by the field size map since the 95% confidence interval did not overlap zero, while the association of higher agricultural value with lower field sizes was not robust to uncertainty (Table 1). The random effects of country on field size were found to be mostly robust, though some countries had significantly more uncertainty in their effects when uncertainty was considered (Fig 3). When we raised our definition of deforestation to require that a higher proportion of sub-cells be deforested for a cell to be considered deforested, our sample size decreased dramatically, leading to an increase in standard errors. The effect sizes of protected area and agricultural value remained however consistent up to relatively high thresholds of 10% deforestation for a cell to be considered deforested (Figure C in S1 File), which was the threshold over which our sample size approximated very low values (Figure D in S1 File). This shows that our results are robust to changes in our definition of deforestation.

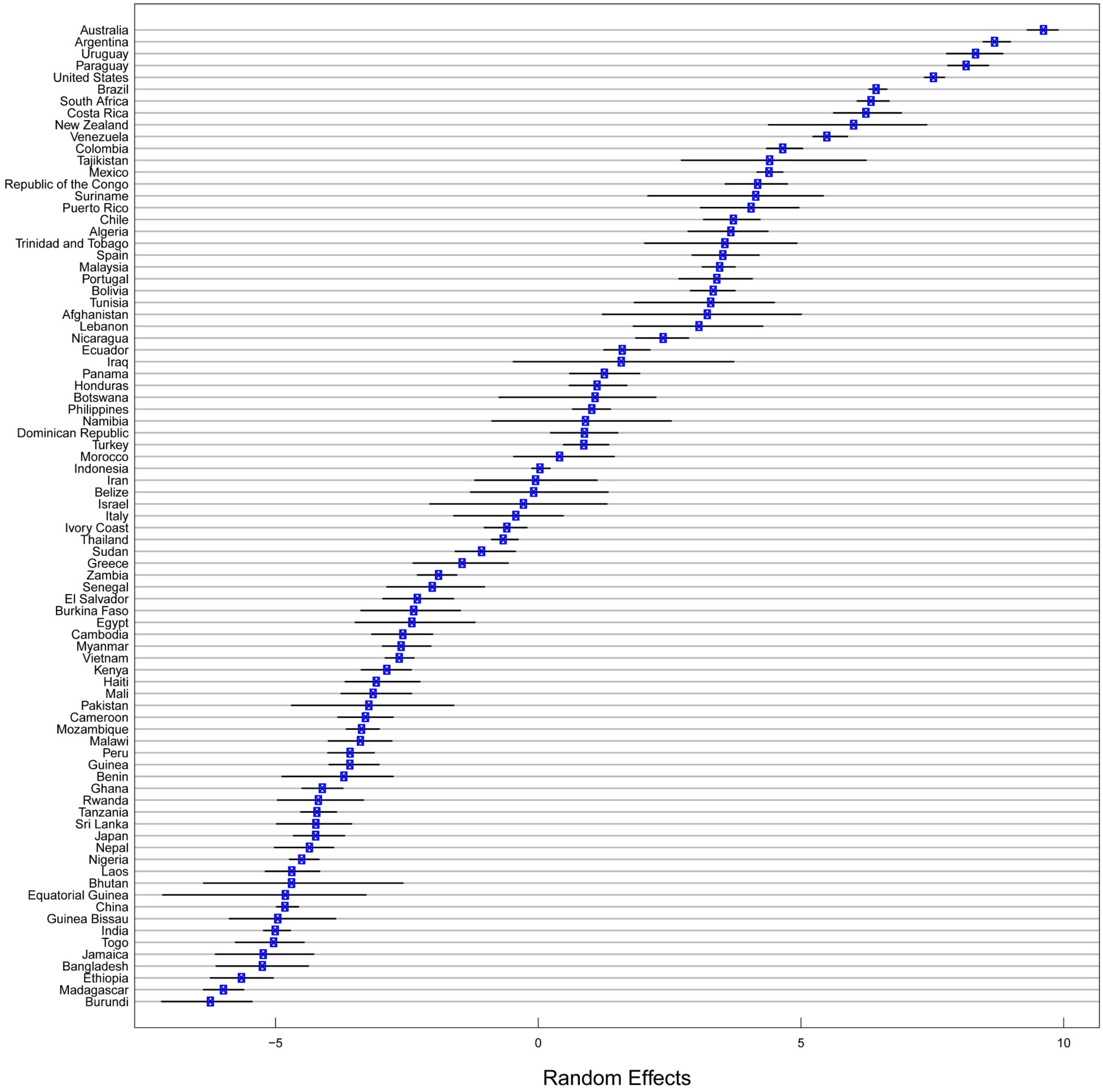

Fig 3. Random intercepts by country indicating the association between field size and deforestation within each country. Error bars are 95% confidence intervals taken from the robustness analysis bootstrap. Positive random effects indicate an associated between large field sizes and deforestation.

https://doi.org/10.1371/journal.pone.0209918.g003

Discussion

The main results of our study are that, first, the country in which deforestation occurs plays the largest role in determining the field size associated with that deforestation. Second, within countries, small field sizes tend to be associated with protected areas and larger field sizes tend to replace forests in areas that have lower agricultural value, although these latter results have considerably less explanatory power. These results may help provide a better understanding of the actors behind deforestation both within and between countries.

The finding that small field sizes are linked to deforestation in protected areas suggests that the actors behind deforestation in these areas are more likely smallholders than large agri-businesses or large family operations. One explanation for this is that it could be easier for smallholders than large agri-businesses or family operations to avoid regulations and encroach on protected areas without repercussions from local governments, which could point towards slash and burn farming or subsistence itinerary agriculture. Further research could look into evaluating the nature and origin of these encroachments by small fields. For instance, knowing whether these results are symptomatic of the displacement of smallholder farmers from large scale concessions or of an increasing scarcity of land that pushes smallholders to protected areas—pointing towards indirect land use change or leakage [30]—would be useful for conservation management. The explanations of why small field sizes are associated to protected areas are however likely to find varying degrees of support depending on the region and agricultural system, requiring further and extensive on-the-ground verification.

Most of the countries with the greatest field sizes behind deforestation were in the Americas and Oceania (in which Australia dominates in terms of contribution to the dataset, Fig 3), which reflects both a high level of development and a dominance of large operations in agriculture and agricultural expansion. However, countries known to be experiencing rapid expansion in agri-business in the period of study, such as Malaysia and Indonesia, also had medium to large field sizes associated with deforestation (Fig 3). These field sizes were behind a sizable proportion of deforestation in other countries in SE Asia and the Americas (Fig 2 and Figure E in S1 File that show the breakdown by countries), pointing towards oil palm agri-businesses in Southeast Asia as major actors behind deforestation by taking advantage of large agricultural concessions [31]. Our results agree with previous studies on patch size of deforestation in the Amazon in which smaller patch sizes (~0.5ha) were observed in Ecuador than in Brazil (~15.6 ha) [32]. In addition, previous studies have found a high proportion of small field sizes (<10 ha) behind deforestation in Western and Central Africa (~ 90%) in 2001, with this proportion being medium (~65% in SE Asia) and low in South America (~40%) [33]. Our results also resonate with a global study identifying higher rates of deforestation by large agribusinesses in Latin America and Southeast Asia, as opposed to Africa where the main drivers relate to smallholders and shifting cultivation [18].

Our results further point towards a link between deforestation to large field sizes and areas of low agricultural value, which could point towards economies of scale and international investment into land reserve areas [34]. Less valuable land presents lower human disturbance and higher biodiversity value, turning large, distant concessions to agri-businesses into a large threat to biodiversity, especially since 34% of large concessions occur at the expense of remote forests [35]. These results suggest that a wide range of actors associated with large field sizes are contributing to deforestation for agriculture in these areas. Thus, our results in combination suggest a narrative of large concessions in areas of less value displacing subsistence farmers towards protected areas. This narrative is however hard to test given the complexities in the interactions between large concessions and subsistence farmers, their potential cooperation and the sequence over time of their operations [36]. In reality, the displacement effects of large concessions are complex and cannot be assessed from global datasets, requiring on-the-ground analyses on a case by case basis.

Among countries with the smallest field size are both countries that are less developed, like Bangladesh or Madagascar, and countries that are more developed but not associated with large agri-business development, such as China and Japan, making again a case for cultural-institutional factors dominating income and level of development when explaining deforestation (Fig 3).

This study presents several limitations, chiefly associated with the uncertainty inherent to the datasets employed. Among this, the main source of uncertainty resides in the field size

dataset that involves crowdsourcing and interpolation across space [19]. The map was created using a qualitative ranking system based on visual inspection and categorization of field size in satellite photographs, rather than a quantitative measurement of field areas [19]. To try to mitigate this situation we employed a robustness analysis using the original expert data used to validate the field size map. Employing bootstrapping methods to modify the datasets showed, however, that the signals we found at the country level and protected area encroachment by small field sizes were robust to uncertainty.

The dataset on deforestation [5], while quite a fine scale map, is known to have difficulties distinguishing between natural forests and tree plantations. This also brings uncertainties due to its definition of deforestation as loss of vegetation taller than 5 meters. Some of the identified deforestation could thus include replacement of trees in tree plantations such as those for oil palm. However we are not aware of global maps of tree crops distribution to correct the original deforestation maps, making this an inherent limitation of the analysis. Other sources of uncertainty are present in the datasets used to create the predictor for agricultural value. This variable was based on potential yields and prices that were later attributed to the most common crop in the cell. This is problematic as the most common crop in the cell is not necessarily the crop that is causing the deforestation. In addition, the distribution of the crops was created using satellite images and ground-based agricultural census statistics [37] which sometimes aggregated crops across large areas and a large periods of time around the year 2000. While this aggregation increases uncertainty and could explain the weaker effect of this variable, to date there are no available accurate high resolution maps of the distributions of major crops [38]. Similarly, there are no maps of capital inputs, technology or level of mechanization of production across the tropics, preventing us for controlling for these factors in production costs. Future research should thus leverage on new maps generated by crowdsourcing and satellite images of increasing resolution to better understand agricultural value across space. Another limitation with the agricultural value variable is that deforestation does not respond to the rents generated by a single crop but by the expected range of benefits from multiple sequential economic activities (e.g. in Latin America deforestation may follow a sequence of initial logging, pasture for cattle ranching and later crops such as maize and soybean). Future research could thus look into partitioning deforestation due to each economic activity [39].

Relatedly, we are using deforestation data from 2000–2001 associated to field size data observed for the year 2005. Our rationale was to use deforestation data as close in time as possible to the agricultural data and to have several years between deforestation and field size estimation for agricultural fields to have time to be established and identified. Identification of agricultural fields four years since deforestation would certainly be possible for annual herbaceous crops and even tree crops like oil palm that require only two to three years to start production [40]. This time gap introduces however a limitation as the field sizes may have changed during the years since deforestation to field size observation. Although rapid changes of field sizes would not be expected in such a short period of time, some exceptions could be due to slash and burn farming and other forms of shifting cultivation that may occur and shift rapidly, leaving the land to be used by other actors. Such rapid turnaround is, however, not so common. Although a large fraction of plots would change crop annually through rotation, the cycle for slash and burn agriculture typically involves multiple years of growing crops (2–4 years) with the nutrients in the soil after conversion taking 2–3 years to peak and followed by a longer fallow cycle [41]. Hence, we would expect that our four year time lag from conversion to field size observation would be able to capture most swidden farmers that performed the conversion towards the end of their cultivation years or the beginning of their fallow period. One reason, however, in which field size and actors could change rapidly may be due to new regulations. For instance, the large agricultural fields in the Brazilian Amazon were divided

under the Land Reform from a size of thousands of hectares into properties of up to 100 hectares [42]. As a result, what would seem to be deforestation from small field sizes would in reality be due to large-scale farming.

Future work should also focus on updating the time period of analysis as soon as more recent field size maps and agricultural maps are available. In addition, considering only one year of deforestation is not ideal. Future work would ideally focus on multiple years of deforestation and multiple layers of field sizes once these datasets become available. The role of large field sizes in deforestation would be even more apparent in recent years (the field size map is from 2005) given intercontinental knowledge transfer innovating deforestation frontier crops to other continents. This is exemplified by large agri-businesses oil palm expansion in Africa [43] and in Latin America [44] including the Peruvian Amazon [45] or soybean from Latin America into Southern African savannah and dry forests [46].

Although field size is known to correlate with farm size, income and level of mechanization [12–14], large field sizes may not always be indicative of large agri-businesses, as this will depend on the agricultural system and crop considered which will itself vary depending on the country. For instance, a large field size in Southeast Asia may be indicative of an oil palm agribusiness company but it may mean large family farm operations in countries such as Paraguay. These idiosyncrasies make difficult to translate field sizes into actual actor typologies but nonetheless field sizes could represent a step forward into gaining insight of potential actors in each region. We nonetheless acknowledge that our analysis is only a small step towards identifying actors behind deforestation and we are still far from identifying, at the pantropical level, the distribution of specific actors with certainty. This highlights the need for map sharing of agribusiness locations and agricultural concessions by local government or by the companies themselves. This information is currently only available for a few countries.

Despite these limitations, our analysis could help understand agricultural field sizes within deforested areas, which can in turn help to plan biodiversity conservation interventions. For instance, for countries where the analysis shows that small field sizes are associated with deforestation, such as the case of Africa and areas of Southeast Asia, payments for ecosystem services, Reducing Emissions from Deforestation and forest Degradation (REDD+) projects, smallholder certification schemes (e.g. existing for coffee and cacao) or alternative livelihoods may be suitable interventions. On the other hand, associations between large field sizes in regions such as Latin America and Southeast Asia may point towards commodity crops such as soybean and sugarcane or oil palm and rubber respectively. In these situations, knowing that large fields of these cash crops may present with high opportunity costs difficult to meet through payments for ecosystem services [47, 48], international consumers' pressure for environmental performance that can translate into zero-deforestation commitments by agri-businesses [49] can be more effective conservation interventions to help balance tropical and subtropical conservation and agricultural production.

The analyses developed here are a small step forward in the characterization of agricultural field sizes behind deforestation with an ultimate end goal to contributing to the identification of the actors behind deforestation across the tropics. While the study revealed the connection between protected areas and deforestation by small field sizes and the influence of country and regional characteristics on field sizes spatially associated to deforestation, more accurate and detailed global datasets have the potential to improve this type of analysis. Increasing our understanding of who drives deforestation will help in turn devise better land-use strategies to contribute to biodiversity conservation, food security, climate resilience and ecosystem service provision.

Supporting information

S1 File. Including Supplementary Methods, Table A, Table B and Figure A, Figure B, Figure C, Figure D and Figure E.
(DOCX)

S1 Table. Original dataset.
(CSV)

Acknowledgments

We thank Steffen Fritz and colleagues for sharing their raw validation data and training images when developing the field size map.

Author Contributions

Conceptualization: Doan K. D. Dang, Luis R. Carrasco.

Data curation: Luis R. Carrasco.

Formal analysis: Doan K. D. Dang, Amy C. Patterson.

Methodology: Amy C. Patterson, Luis R. Carrasco.

Project administration: Luis R. Carrasco.

Resources: Luis R. Carrasco.

Supervision: Luis R. Carrasco.

Validation: Doan K. D. Dang, Amy C. Patterson, Luis R. Carrasco.

Visualization: Doan K. D. Dang.

Writing – original draft: Doan K. D. Dang, Luis R. Carrasco.

Writing – review & editing: Amy C. Patterson, Luis R. Carrasco.

References

1. Myers N, Mittermeier RA, Mittermeier CG, da Fonseca GAB, Kent J. Biodiversity hotspots for conservation priorities. Nature. 2000; 403(6772):853–8. http://www.nature.com/nature/journal/v403/n6772/suppinfo/403853a0_S1.html. https://doi.org/10.1038/35002501 PMID: 10706275
2. Sheil D. How plants water our planet: advances and imperatives. Trends in plant science. 2014; 19 (4):209–11. https://doi.org/10.1016/j.tplants.2014.01.002 PMID: 24698708
3. Gibbs HK, Brown S, Niles JO, Foley JA. Monitoring and estimating tropical forest carbon stocks: making REDD a reality. Environmental Research Letters. 2007; 2(4):045023.
4. DeFries RS, Foley JA, Asner GP. Land-use choices: Balancing human needs and ecosystem function. Frontiers in Ecology and the Environment. 2004; 2(5):249–57.
5. Hansen MC, Potapov PV, Moore R, Hancher M, Turubanova SA, Tyukavina A, et al. High-Resolution Global Maps of 21st-Century Forest Cover Change. Science. 2013;342. https://doi.org/10.1126/science.1244693 PMID: 24233722
6. Wilcove DS, Giam X, Edwards DP, Fisher B, Koh LP. Navjot's nightmare revisited: logging, agriculture, and biodiversity in Southeast Asia. Trends in Ecology & Evolution. 2013; 28(9):531–40.
7. Carrasco LR, Le Nghiem TP, Chen Z, Barbier EB. Unsustainable development pathways caused by tropical deforestation. Science advances. 2017; 3(7):e1602602. https://doi.org/10.1126/sciadv.1602602 PMID: 28706988
8. Gibbs H, Ruesch A, Achard F, Clayton M, Holmgren P, Ramankutty N, et al. Tropical forests were the primary sources of new agricultural land in the 1980s and 1990s. Proc Natl Acad Sci U S A. 2010; 107 (70150537-3c4a-be3e-97ae-be3107c1b3be):16732–9. https://doi.org/10.1073/pnas.0910275107 PMID: 20807750

9. Graesser J, Aide TM, Grau HR, Ramankutty N. Cropland/pastureland dynamics and the slowdown of deforestation in Latin America. Environ Res Lett. 2015; 10(3):034017.

10. Mayaux P, Pekel J-F, Desclée B, Donnay F, Lupi A, Achard F, et al. State and evolution of the African rainforests between 1990 and 2010. Philosophical Transactions of the Royal Society of London B: Biological Sciences. 2013; 368(1625):20120300. https://doi.org/10.1098/rstb.2012.0300 PMID: 23878331

11. FAO GR. The economic lives of smallholder farmers: An analysis based on household data from nine countries. Food and Agriculture Organization of the United Nations, Rome, Italy. 2015.

12. Levin G. Farm size and landscape composition in relation to landscape changes in Denmark. Geografisk Tidsskrift-Danish Journal of Geography. 2006; 106(2):45–59.

13. Berry RA. Farm size distribution, income distribution, and the efficiency of agricultural production: Colombia. The American Economic Review. 1972:403–8.

14. Kuemmerle T, Erb K, Meyfroidt P, Müller D, Verburg PH, Estel S, et al. Challenges and opportunities in mapping land use intensity globally. Current Opinion in Environmental Sustainability. 2013; 5(5):484–93. https://doi.org/10.1016/j.cosust.2013.06.002 PMID: 24143157

15. Rosa I, SOUZA C, Ewers RM. Changes in size of deforested patches in the Brazilian Amazon. Conservation Biology. 2012; 26(5):932–7. https://doi.org/10.1111/j.1523-1739.2012.01901.x PMID: 22827325

16. Godar J, Gardner TA, Tizado EJ, Pacheco P. Actor-specific contributions to the deforestation slowdown in the Brazilian Amazon. Proceedings of the National Academy of Sciences. 2014; 111(43):15591–6.

17. Lee JSH, Abood S, Ghazoul J, Barus B, Obidzinski K, Koh LP. Environmental Impacts of Large-Scale Oil Palm Enterprises Exceed that of Smallholdings in Indonesia. Conservation Letters. 2014; 7(1):25–33.

18. Curtis PG, Slay CM, Harris NL, Tyukavina A, Hansen MC. Classifying drivers of global forest loss. Science. 2018; 361(6407):1108–11. https://doi.org/10.1126/science.aau3445 PMID: 30213911

19. Fritz S, See L, McCallum I, You L, Bun A, Moltchanova E, et al. Mapping global cropland and field size. Global Change Biology. 2015.

20. Symes WS, Rao M, Mascia MB, Carrasco LR. Why do we lose protected areas? Factors influencing protected area downgrading, downsizing and degazettement in the tropics and subtropics. Global Change Biology. 2015.

21. WDPA Consortium. World database on protected areas. World Conservation Union and UNEP-World Conservation Monitoring Centre, New York, New York, USA. 2018.

22. Brun C, Cook AR, Lee JSH, Wich SA, Koh LP, Carrasco LR. Analysis of deforestation and protected area effectiveness in Indonesia: A comparison of Bayesian spatial models. Global Environmental Change. 2015; 31:285–95.

23. Nelson A. Accessibility model and population estimates. background paper and digital files prepared for the World Development Report. 2009.

24. CIESIN, CIAT. Gridded Population of the World, Version 3 (GPWv3). Palisades, NY: CIESIN, Columbia University; 2005.

25. Phelps J, Carrasco LR, Webb EL, Koh LP, Pascual U. Agricultural intensification escalates future conservation costs. Proceedings of the National Academy of Sciences. 2013. https://doi.org/10.1073/pnas.1220070110 PMID: 23589860

26. Etongo D, Djenontin INS, Kanninen M, Fobissie K, Korhonen-Kurki K, Djoudi H. Land tenure, asset heterogeneity and deforestation in Southern Burkina Faso. Forest Policy and Economics. 2015; 61:51–8. http://dx.doi.org/10.1016/j.forpol.2015.08.006.

27. Pinheiro J, Bates D, DebRoy S, Sarkar D, inventorsnlme: Linear and nonlinear mixed effects models2006.

28. R Development Core Team. R: A Language and Environment for Statistical Computing. R Foundation for Statistical Computing. Vienna, Austria. http://www.R-project.org/. 2015.

29. Burnham KP, Anderson DR. Model selection and multi-model inference: a practical information-theoretic approach: Springer Verlag; 2002.

30. Oliveira PJC, Asner GP, Knapp DE, Almeyda A, Galvan-Gildemeister R, Keene S, et al. Land-Use Allocation Protects the Peruvian Amazon. Science. 2007; 317(3c16300d-e2a3-9557-7c81-be5d56c0e65f). https://doi.org/10.1126/science.1146324 PMID: 17690260

31. Davis KF, Yu K, Rulli MC, Pichdara L, D'Odorico P. Accelerated deforestation driven by large-scale land acquisitions in Cambodia. Nature Geosci. 2015; 8(10):772–5. https://doi.org/10.1038/ngeo2540 http://www.nature.com/ngeo/journal/v8/n10/abs/ngeo2540.html#supplementary-information.

32. Kalamandeen M, Gloor E, Mitchard E, Quincey D, Ziv G, Spracklen D, et al. Pervasive rise of small-scale deforestation in Amazonia. Scientific Reports. 2018; 8(1):1600. https://doi.org/10.1038/s41598-018-19358-2 PMID: 29371623

33. Austin KG, González-Roglich M, Schaffer-Smith D, Schwantes AM, Swenson JJ. Trends in size of tropical deforestation events signal increasing dominance of industrial-scale drivers. Environ Res Lett. 2017; 12(5):054009.
34. Meyfroidt P, Rudel TK, Lambin EF. Forest transitions, trade, and the global displacement of land use. Proc Natl Acad Sci U S A. 2010; 107(49):20917–22. https://doi.org/10.1073/pnas.1014773107 PMID: 21078977
35. Messerli P, Giger M, Dwyer MB, Breu T, Eckert S. The geography of large-scale land acquisitions: Analysing socio-ecological patterns of target contexts in the global South. Appl Geogr. 2014; 53:449–59.
36. Papworth S, Rao M, Oo MM, Latt KT, Tizard R, Pienkowski T, et al. The impact of gold mining and agricultural concessions on the tree cover and local communities in northern Myanmar. Scientific Reports. 2017; 7.
37. Ramankutty N, Evan AT, Monfreda C, Foley JA. Farming the planet: 1. Geographic distribution of global agricultural lands in the year 2000. Global Biogeochemical Cycles. 2008; 22(1).
38. See L, Fritz S, You L, Ramankutty N, Herrero M, Justice C, et al. Improved global cropland data as an essential ingredient for food security. Global Food Security. 2015; 4:37–45. http://dx.doi.org/10.1016/j.gfs.2014.10.004.
39. Cederberg C, Persson UM, Neovius K, Molander S, Clift R. Including Carbon Emissions from Deforestation in the Carbon Footprint of Brazilian Beef. Environ Sci Technol. 2011; 45(5):1773–9. https://doi.org/10.1021/es103240z PMID: 21280649
40. Corley RHV, Tinker P. The oil palm: John Wiley & Sons; 2008.
41. Kleinman P, Pimentel D, Bryant RB. The ecological sustainability of slash-and-burn agriculture. Agriculture, Ecosystems & Environment. 1995; 52(2–3):235–49.
42. Pacheco P. Agrarian reform in the Brazilian Amazon: its implications for land distribution and deforestation. World Dev. 2009; 37(8):1337–47.
43. Carrasco L, Larrosa C, Milner-Gulland E, Edwards D. A double-edged sword for tropical forests. Science. 2014; 346(6205):38–40. https://doi.org/10.1126/science.1256685 PMID: 25278600
44. Furumo PR, Aide TM. Characterizing commercial oil palm expansion in Latin America: land use change and trade. Environ Res Lett. 2017; 12(2):024008.
45. Gutierrez-Velez VH, DeFries R. Annual multi-resolution detection of land cover conversion to oil palm in the Peruvian Amazon. Remote Sensing of Environment. 2013; 129:154–67. https://doi.org/10.1016/j.rse.2012.10.033 WOS:000315308300014.
46. Gasparri NI, Kuemmerle T, Meyfroidt P, Waroux Y, Kreft H. The emerging soybean production frontier in Southern Africa: Conservation challenges and the role of south-south telecouplings. Conservation Letters. 2016; 9(1):21–31.
47. Fisher B, Edwards DP, Giam X, Wilcove DS. The high costs of conserving Southeast Asia's lowland rainforests. Frontiers in Ecology and the Environment. 2011; 9:329–34. https://doi.org/10.1890/100079
48. Carrasco LR, Webb EL, Symes WS, Koh LP, Sodhi NS. Global economic trade-offs between wild nature and tropical agriculture. PLoS Biology. 2017; 15(7):e2001657. https://doi.org/10.1371/journal.pbio.2001657 PMID: 28732022
49. Pirard R, Gnych S, Pacheco P, Lawry S. Zero-deforestation commitments in Indonesia: Governance challenges. Center for International Forestry Research (CIFOR), Bogor, Indonesia, 2015.
